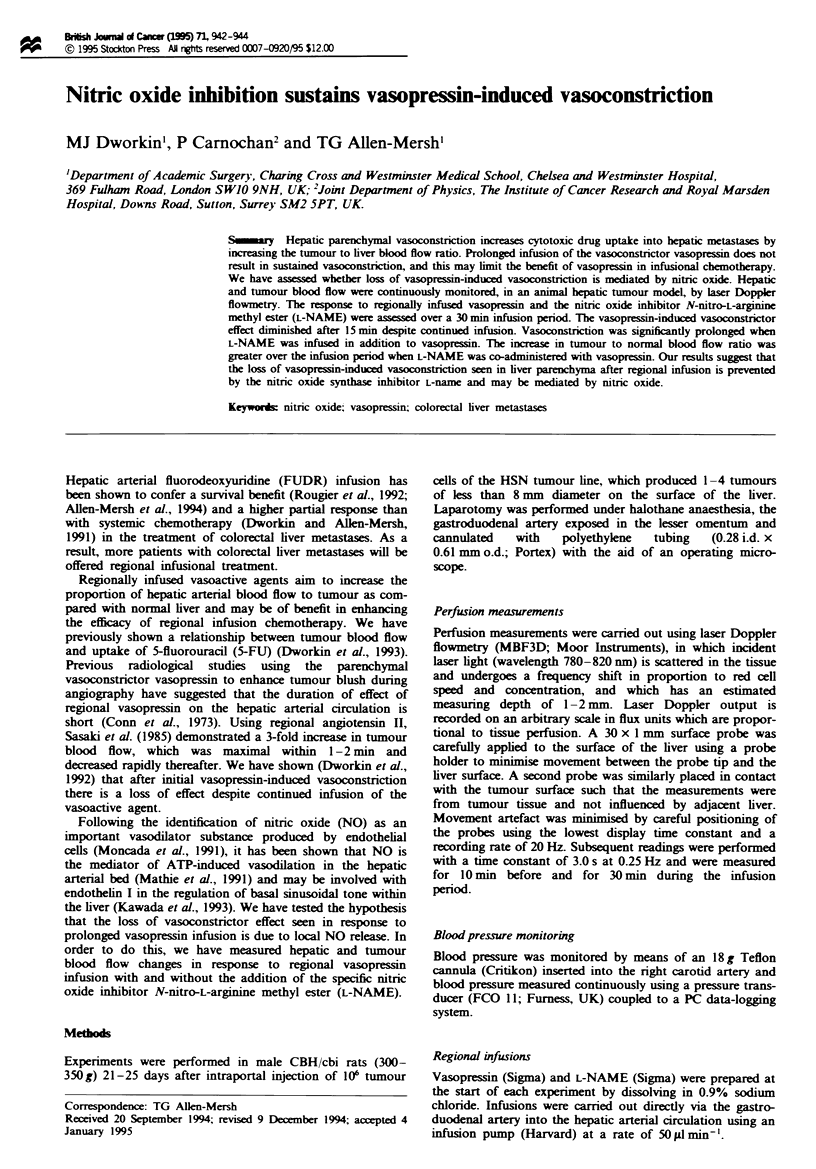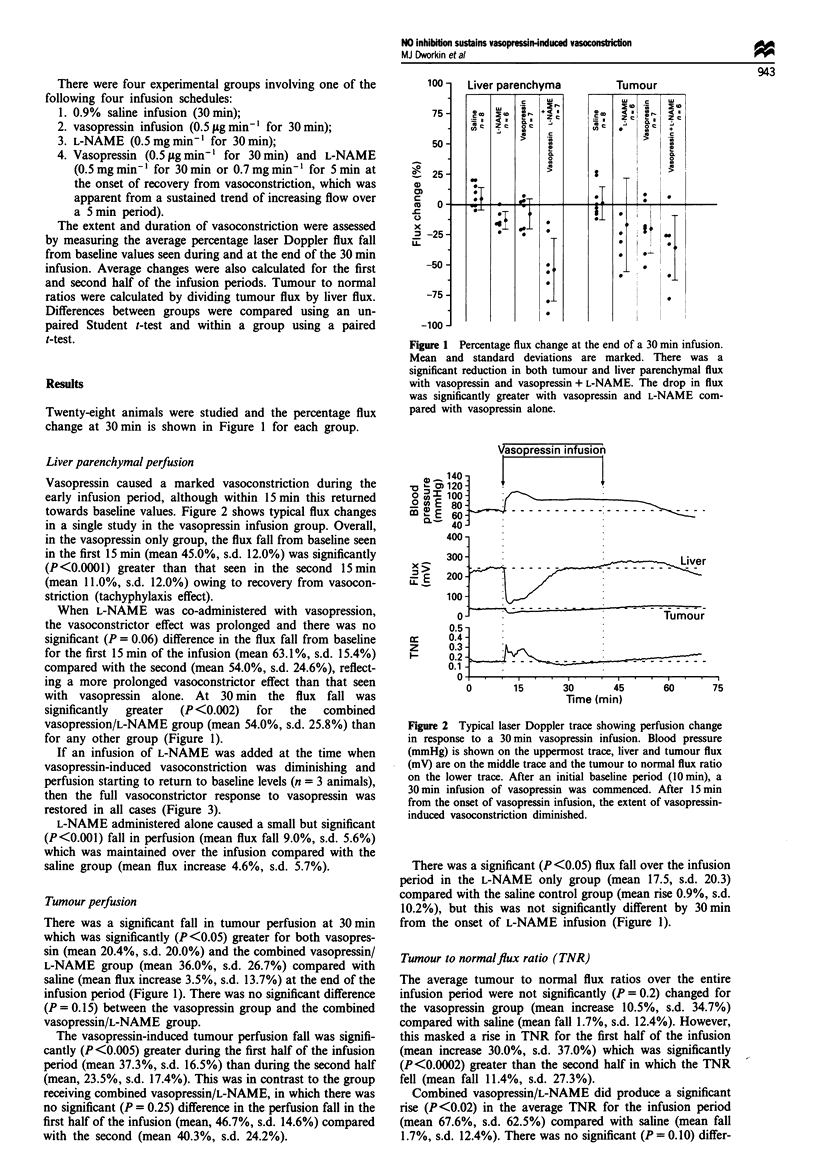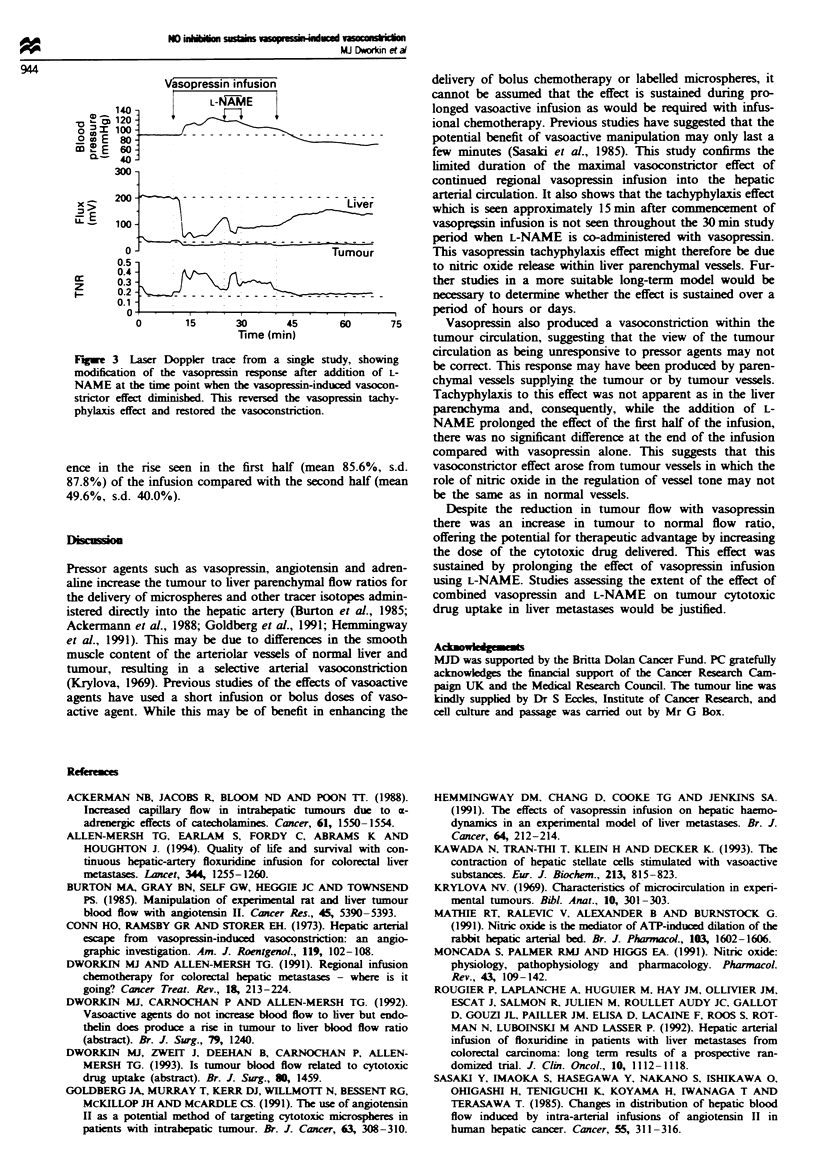# Nitric oxide inhibition sustains vasopressin-induced vasoconstriction.

**DOI:** 10.1038/bjc.1995.182

**Published:** 1995-05

**Authors:** M. J. Dworkin, P. Carnochan, T. G. Allen-Mersh

**Affiliations:** Department of Academic Surgery, Charing Cross and Westminster Medical School, Chelsea and Westminster Hospital, London, UK.

## Abstract

Hepatic parenchymal vasoconstriction increases cytotoxic drug uptake into hepatic metastases by increasing the tumour to liver blood flow ratio. Prolonged infusion of the vasoconstrictor vasopressin does not result in sustained vasoconstriction, and this may limit the benefit of vasopressin in infusional chemotherapy. We have assessed whether loss of vasopressin-induced vasoconstriction is mediated by nitric oxide. Hepatic and tumour blood flow were continuously monitored, in an animal hepatic tumour model, by laser Doppler flowmetry. The response to regionally infused vasopressin and the nitric oxide inhibitor N-nitro-L-arginine methyl ester (L-NAME) were assessed over a 30 min infusion period. The vasopressin-induced vasoconstrictor effect diminished after 15 min despite continued infusion. Vasoconstriction was significantly prolonged when L-NAME was infused in addition to vasopressin. The increase in tumour to normal blood flow ratio was greater over the infusion period when L-NAME was co-administered with vasopressin. Our results suggest that the loss of vasopressin-induced vasoconstriction seen in liver parenchyma after regional infusion is prevented by the nitric oxide synthase inhibitor L-name and may be mediated by nitric oxide.


British Journal of Cancer (1995) 71, 942-944

© 1995 Stockton Press All rights reserved 0007-0920/95 $12.00

### Nitric oxide inhibition sustains vasopressin-induced vasoconstriction

MJ Dworkin[1], P Carnochan[2] and TG Allen-Mersh[1]

[1]*Department of Academic Surgery, Charing Cross and Westminster Medical School, Chelsea and Westminster Hospital, 369 Fulham Road, London SW10 9NH, UK;* [2]*Joint Department of Physics, The Institute of Cancer Research and Royal Marsden Hospital, Downs Road, Sutton, Surrey SM2 5PT, UK.*

**Summary** Hepatic parenchymal vasoconstriction increases cytotoxic drug uptake into hepatic metastases by increasing the tumour to liver blood flow ratio. Prolonged infusion of the vasoconstrictor vasopressin does not result in sustained vasoconstriction, and this may limit the benefit of vasopressin in infusional chemotherapy. We have assessed whether loss of vasopressin-induced vasoconstriction is mediated by nitric oxide. Hepatic and tumour blood flow were continuously monitored, in an animal hepatic tumour model, by laser Doppler flowmetry. The response to regionally infused vasopressin and the nitric oxide inhibitor *N*-nitro-L-arginine methyl ester (L-NAME) were assessed over a 30 min infusion period. The vasopressin-induced vasoconstrictor effect diminished after 15 min despite continued infusion. Vasoconstriction was significantly prolonged when L-NAME was infused in addition to vasopressin. The increase in tumour to normal blood flow ratio was greater over the infusion period when L-NAME was co-administered with vasopressin. Our results suggest that the loss of vasopressin-induced vasoconstriction seen in liver parenchyma after regional infusion is prevented by the nitric oxide synthase inhibitor L-name and may be mediated by nitric oxide.

**Keywords:** nitric oxide; vasopressin; colorectal liver metastases

Hepatic arterial fluorodeoxyuridine (FUDR) infusion has been shown to confer a survival benefit (Rougier *et al.*, 1992; Allen-Mersh *et al.*, 1994) and a higher partial response than with systemic chemotherapy (Dworkin and Allen-Mersh, 1991) in the treatment of colorectal liver metastases. As a result, more patients with colorectal liver metastases will be offered regional infusional treatment.

Regionally infused vasoactive agents aim to increase the proportion of hepatic arterial blood flow to tumour as compared with normal liver and may be of benefit in enhancing the efficacy of regional infusion chemotherapy. We have previously shown a relationship between tumour blood flow and uptake of 5-fluorouracil (5-FU) (Dworkin *et al.*, 1993). Previous radiological studies using the parenchymal vasoconstrictor vasopressin to enhance tumour blush during angiography have suggested that the duration of effect of regional vasopressin on the hepatic arterial circulation is short (Conn *et al.*, 1973). Using regional angiotensin II, Sasaki *et al.* (1985) demonstrated a 3-fold increase in tumour blood flow, which was maximal within 1-2 min and decreased rapidly thereafter. We have shown (Dworkin *et al.*, 1992) that after initial vasopressin-induced vasoconstriction there is a loss of effect despite continued infusion of the vasoactive agent.

Following the identification of nitric oxide (NO) as an important vasodilator substance produced by endothelial cells (Moncada *et al.*, 1991), it has been shown that NO is the mediator of ATP-induced vasodilation in the hepatic arterial bed (Mathie *et al.*, 1991) and may be involved with endothelin I in the regulation of basal sinusoidal tone within the liver (Kawada *et al.*, 1993). We have tested the hypothesis that the loss of vasoconstrictor effect seen in response to prolonged vasopressin infusion is due to local NO release. In order to do this, we have measured hepatic and tumour blood flow changes in response to regional vasopressin infusion with and without the addition of the specific nitric oxide inhibitor *N*-nitro-L-arginine methyl ester (L-NAME).

### Methods

Experiments were performed in male CBH/cbi rats (300-350 *g*) 21-25 days after intraportal injection of $10^6$ tumour cells of the HSN tumour line, which produced 1-4 tumours of less than 8 mm diameter on the surface of the liver. Laparotomy was performed under halothane anaesthesia, the gastroduodenal artery exposed in the lesser omentum and cannulated with polyethylene tubing (0.28 i.d. × 0.61 mm o.d.; Portex) with the aid of an operating microscope.

### *Perfusion measurements*

Perfusion measurements were carried out using laser Doppler flowmetry (MBF3D; Moor Instruments), in which incident laser light (wavelength 780-820 nm) is scattered in the tissue and undergoes a frequency shift in proportion to red cell speed and concentration, and which has an estimated measuring depth of 1-2 mm. Laser Doppler output is recorded on an arbitrary scale in flux units which are proportional to tissue perfusion. A 30 × 1 mm surface probe was carefully applied to the surface of the liver using a probe holder to minimise movement between the probe tip and the liver surface. A second probe was similarly placed in contact with the tumour surface such that the measurements were from tumour tissue and not influenced by adjacent liver. Movement artefact was minimised by careful positioning of the probes using the lowest display time constant and a recording rate of 20 Hz. Subsequent readings were performed with a time constant of 3.0 s at 0.25 Hz and were measured for 10 min before and for 30 min during the infusion period.

### *Blood pressure monitoring*

Blood pressure was monitored by means of an 18 *g* Teflon cannula (Critikon) inserted into the right carotid artery and blood pressure measured continuously using a pressure transducer (FCO 11; Furness, UK) coupled to a PC data-logging system.

### *Regional infusions*

Vasopressin (Sigma) and L-NAME (Sigma) were prepared at the start of each experiment by dissolving in 0.9% sodium chloride. Infusions were carried out directly via the gastroduodenal artery into the hepatic arterial circulation using an infusion pump (Harvard) at a rate of $50\,\mu l\,min^{-1}$.

Correspondence: TG Allen-Mersh
Received 20 September 1994; revised 9 December 1994; accepted 4 January 1995

There were four experimental groups involving one of the following four infusion schedules:

1. 0.9% saline infusion (30 min);

2. vasopressin infusion (0.5 μg min$^{-1}$ for 30 min);

3. L-NAME (0.5 mg min$^{-1}$ for 30 min);

4. Vasopressin (0.5 μg min$^{-1}$ for 30 min) and L-NAME (0.5 mg min$^{-1}$ for 30 min or 0.7 mg min$^{-1}$ for 5 min at the onset of recovery from vasoconstriction, which was apparent from a sustained trend of increasing flow over a 5 min period).

The extent and duration of vasoconstriction were assessed by measuring the average percentage laser Doppler flux fall from baseline values seen during and at the end of the 30 min infusion. Average changes were also calculated for the first and second half of the infusion periods. Tumour to normal ratios were calculated by dividing tumour flux by liver flux. Differences between groups were compared using an unpaired Student *t*-test and within a group using a paired *t*-test.

### Results

Twenty-eight animals were studied and the percentage flux change at 30 min is shown in Figure 1 for each group.

### *Liver parenchymal perfusion*

Vasopressin caused a marked vasoconstriction during the early infusion period, although within 15 min this returned towards baseline values. Figure 2 shows typical flux changes in a single study in the vasopressin infusion group. Overall, in the vasopressin only group, the flux fall from baseline seen in the first 15 min (mean 45.0%, s.d. 12.0%) was significantly ($P<0.0001$) greater than that seen in the second 15 min (mean 11.0%, s.d. 12.0%) owing to recovery from vasoconstriction (tachyphylaxis effect).

When L-NAME was co-administered with vasopression, the vasoconstrictor effect was prolonged and there was no significant ($P = 0.06$) difference in the flux fall from baseline for the first 15 min of the infusion (mean 63.1%, s.d. 15.4%) compared with the second (mean 54.0%, s.d. 24.6%), reflecting a more prolonged vasoconstrictor effect than that seen with vasopressin alone. At 30 min the flux fall was significantly greater ($P<0.002$) for the combined vasopression/L-NAME group (mean 54.0%, s.d. 25.8%) than for any other group (Figure 1).

If an infusion of L-NAME was added at the time when vasopressin-induced vasoconstriction was diminishing and perfusion starting to return to baseline levels ($n = 3$ animals), then the full vasoconstrictor response to vasopressin was restored in all cases (Figure 3).

L-NAME administered alone caused a small but significant ($P<0.001$) fall in perfusion (mean flux fall 9.0%, s.d. 5.6%) which was maintained over the infusion compared with the saline group (mean flux increase 4.6%, s.d. 5.7%).

### *Tumour perfusion*

There was a significant fall in tumour perfusion at 30 min which was significantly ($P<0.05$) greater for both vasopressin (mean 20.4%, s.d. 20.0%) and the combined vasopressin/L-NAME group (mean 36.0%, s.d. 26.7%) compared with saline (mean flux increase 3.5%, s.d. 13.7%) at the end of the infusion period (Figure 1). There was no significant difference ($P = 0.15$) between the vasopressin group and the combined vasopressin/L-NAME group.

The vasopressin-induced tumour perfusion fall was significantly ($P<0.005$) greater during the first half of the infusion period (mean 37.3%, s.d. 16.5%) than during the second half (mean, 23.5%, s.d. 17.4%). This was in contrast to the group receiving combined vasopressin/L-NAME, in which there was no significant ($P = 0.25$) difference in the perfusion fall in the first half of the infusion (mean, 46.7%, s.d. 14.6%) compared with the second (mean 40.3%, s.d. 24.2%).

**Figure 1** Percentage flux change at the end of a 30 min infusion. Mean and standard deviations are marked. There was a significant reduction in both tumour and liver parenchymal flux with vasopressin and vasopressin + L-NAME. The drop in flux was significantly greater with vasopressin and L-NAME compared with vasopressin alone.

**Figure 2** Typical laser Doppler trace showing perfusion change in response to a 30 min vasopressin infusion. Blood pressure (mmHg) is shown on the uppermost trace, liver and tumour flux (mV) are on the middle trace and the tumour to normal flux ratio on the lower trace. After an initial baseline period (10 min), a 30 min infusion of vasopressin was commenced. After 15 min from the onset of vasopressin infusion, the extent of vasopressin-induced vasoconstriction diminished.

There was a significant ($P<0.05$) flux fall over the infusion period in the L-NAME only group (mean 17.5, s.d. 20.3) compared with the saline control group (mean rise 0.9%, s.d. 10.2%), but this was not significantly different by 30 min from the onset of L-NAME infusion (Figure 1).

### *Tumour to normal flux ratio (TNR)*

The average tumour to normal flux ratios over the entire infusion period were not significantly ($P = 0.2$) changed for the vasopressin group (mean increase 10.5%, s.d. 34.7%) compared with saline (mean fall 1.7%, s.d. 12.4%). However, this masked a rise in TNR for the first half of the infusion (mean increase 30.0%, s.d. 37.0%) which was significantly ($P<0.0002$) greater than the second half in which the TNR fell (mean fall 11.4%, s.d. 27.3%).

Combined vasopressin/L-NAME did produce a significant rise ($P<0.02$) in the average TNR for the infusion period (mean 67.6%, s.d. 62.5%) compared with saline (mean fall 1.7%, s.d. 12.4%). There was no significant ($P = 0.10$) differ-

**Figure 3** Laser Doppler trace from a single study, showing modification of the vasopressin response after addition of L-NAME at the time point when the vasopressin-induced vasoconstrictor effect diminished. This reversed the vasopressin tachyphylaxis effect and restored the vasoconstriction.

ence in the rise seen in the first half (mean 85.6%, s.d. 87.8%) of the infusion compared with the second half (mean 49.6%, s.d. 40.0%).

### Discussion

Pressor agents such as vasopressin, angiotensin and adrenaline increase the tumour to liver parenchymal flow ratios for the delivery of microspheres and other tracer isotopes administered directly into the hepatic artery (Burton *et al.*, 1985; Ackermann *et al.*, 1988; Goldberg *et al.*, 1991; Hemmingway *et al.*, 1991). This may be due to differences in the smooth muscle content of the arteriolar vessels of normal liver and tumour, resulting in a selective arterial vasoconstriction (Krylova, 1969). Previous studies of the effects of vasoactive agents have used a short infusion or bolus doses of vasoactive agent. While this may be of benefit in enhancing the delivery of bolus chemotherapy or labelled microspheres, it cannot be assumed that the effect is sustained during prolonged vasoactive infusion as would be required with infusional chemotherapy. Previous studies have suggested that the potential benefit of vasoactive manipulation may only last a few minutes (Sasaki *et al.*, 1985). This study confirms the limited duration of the maximal vasoconstrictor effect of continued regional vasopressin infusion into the hepatic arterial circulation. It also shows that the tachyphylaxis effect which is seen approximately 15 min after commencement of vasopressin infusion is not seen throughout the 30 min study period when L-NAME is co-administered with vasopressin. This vasopressin tachyphylaxis effect might therefore be due to nitric oxide release within liver parenchymal vessels. Further studies in a more suitable long-term model would be necessary to determine whether the effect is sustained over a period of hours or days.

Vasopressin also produced a vasoconstriction within the tumour circulation, suggesting that the view of the tumour circulation as being unresponsive to pressor agents may not be correct. This response may have been produced by parenchymal vessels supplying the tumour or by tumour vessels. Tachyphylaxis to this effect was not apparent as in the liver parenchyma and, consequently, while the addition of L-NAME prolonged the effect of the first half of the infusion, there was no significant difference at the end of the infusion compared with vasopressin alone. This suggests that this vasoconstrictor effect arose from tumour vessels in which the role of nitric oxide in the regulation of vessel tone may not be the same as in normal vessels.

Despite the reduction in tumour flow with vasopressin there was an increase in tumour to normal flow ratio, offering the potential for therapeutic advantage by increasing the dose of the cytotoxic drug delivered. This effect was sustained by prolonging the effect of vasopressin infusion using L-NAME. Studies assessing the extent of the effect of combined vasopressin and L-NAME on tumour cytotoxic drug uptake in liver metastases would be justified.

**Acknowledgements**
MJD was supported by the Britta Dolan Cancer Fund. PC gratefully acknowledges the financial support of the Cancer Research Campaign UK and the Medical Research Council. The tumour line was kindly supplied by Dr S Eccles, Institute of Cancer Research, and cell culture and passage was carried out by Mr G Box.

### References

ACKERMAN NB, JACOBS R, BLOOM ND AND POON TT. (1988). Increased capillary flow in intrahepatic tumours due to α-adrenergic effects of catecholamines. *Cancer*, **61**, 1550-1554.

ALLEN-MERSH TG, EARLAM S, FORDY C, ABRAMS K AND HOUGHTON J. (1994). Quality of life and survival with continuous hepatic-artery floxuridine infusion for colorectal liver metastases. *Lancet*, **344**, 1255-1260.

BURTON MA, GRAY BN, SELF GW, HEGGIE JC AND TOWNSEND PS. (1985). Manipulation of experimental rat and liver tumour blood flow with angiotensin II. *Cancer Res.*, **45**, 5390-5393.

CONN HO, RAMSBY GR AND STORER EH. (1973). Hepatic arterial escape from vasopressin-induced vasoconstriction: an angiographic investigation. *Am. J. Roentgenol.*, **119**, 102-108.

DWORKIN MJ AND ALLEN-MERSH TG. (1991). Regional infusion chemotherapy for colorectal hepatic metastases – where is it going? *Cancer Treat. Rev.*, **18**, 213-224.

DWORKIN MJ, CARNOCHAN P AND ALLEN-MERSH TG. (1992). Vasoactive agents do not increase blood flow to liver but endothelin does produce a rise in tumour to liver blood flow ratio (abstract). *Br. J. Surg.*, **79**, 1240.

DWORKIN MJ, ZWEIT J, DEEHAN B, CARNOCHAN P, ALLEN-MERSH TG. (1993). Is tumour blood flow related to cytotoxic drug uptake (abstract). *Br. J. Surg.*, **80**, 1459.

GOLDBERG JA, MURRAY T, KERR DJ, WILLMOTT N, BESSENT RG, McKILLOP JH AND McARDLE CS. (1991). The use of angiotensin II as a potential method of targeting cytotoxic microspheres in patients with intrahepatic tumour. *Br. J. Cancer*, **63**, 308-310.

HEMMINGWAY DM, CHANG D, COOKE TG AND JENKINS SA. (1991). The effects of vasopressin infusion on hepatic haemodynamics in an experimental model of liver metastases. *Br. J. Cancer*, **64**, 212-214.

KAWADA N, TRAN-THI T, KLEIN H AND DECKER K. (1993). The contraction of hepatic stellate cells stimulated with vasoactive substances. *Eur. J. Biochem.*, **213**, 815-823.

KRYLOVA NV. (1969). Characteristics of microcirculation in experimental tumours. *Bibl. Anat.*, **10**, 301-303.

MATHIE RT, RALEVIC V, ALEXANDER B AND BURNSTOCK G. (1991). Nitric oxide is the mediator of ATP-induced dilation of the rabbit hepatic arterial bed. *Br. J. Pharmacol.*, **103**, 1602-1606.

MONCADA S, PALMER RMJ AND HIGGS EA. (1991). Nitric oxide: physiology, pathophysiology and pharmacology. *Pharmacol. Rev.*, **43**, 109-142.

ROUGIER P, LAPLANCHE A, HUGUIER M, HAY JM, OLLIVIER JM, ESCAT J, SALMON R, JULIEN M, ROULLET AUDY JC, GALLOT D, GOUZI JL, PAILLER JM, ELISA D, LACAINE F, ROOS S, ROTMAN N, LUBOINSKI M AND LASSER P. (1992). Hepatic arterial infusion of floxuridine in patients with liver metastases from colorectal carcinoma: long term results of a prospective randomized trial. *J. Clin. Oncol.*, **10**, 1112-1118.

SASAKI Y, IMAOKA S, HASEGAWA Y, NAKANO S, ISHIKAWA O, OHIGASHI H, TENIGUCHI K, KOYAMA H, IWANAGA T AND TERASAWA T. (1985). Changes in distribution of hepatic blood flow induced by intra-arterial infusions of angiotensin II in human hepatic cancer. *Cancer*, **55**, 311-316.